# Efficacy and safety of long-term therapy for high-grade glioma with temozolomide: A meta-analysis

**DOI:** 10.18632/oncotarget.17401

**Published:** 2017-04-24

**Authors:** Weilin Xu, Tao Li, Liansheng Gao, Jingwei Zheng, Anwen Shao, Jianmin Zhang

**Affiliations:** ^1^ Department of Neurosurgery, Second Affiliated Hospital, School of Medicine, Zhejiang University, Hangzhou, Zhejiang, China; ^2^ Brain Research Institute, Zhejiang University, Hangzhou, Zhejiang, China; ^3^ Collaborative Innovation Center for Brain Science, Zhejiang University, Hangzhou, Zhejiang, China

**Keywords:** long-term, glioma, temozolomide, meta-analysis

## Abstract

Further treatments are warranted in preventing recurrence or progression for high-grade glioma (HGG) patients having achieved stable disease with tolerable toxicity after the Stupp regimen (6 cycles of temozolomide). This meta-analysis aims to extensively evaluate the safety, feasibility, and efficacy of long-term therapy with temozolomide (>6 cycles) for these patients. We systematically searched the pubmed, Embase and Chinese Biomedical (CBM) databases using the strategy of combination of free-text words and MeSH terms. The efficacy indicators are hazard ratio (HR) for the pooled analysis of overall survival (OS) and progression free survival (PFS). The safety indicator is risk ratio (RR) for the pooled analysis of adverse effects. Six studies comprising a total number of 396 patients met all inclusion and exclusion criteria were included. No heterogeneity and publication bias were observed across each study. It was found that patients could obtain benefits from long-term administration of temozolomide both in OS (HR 2.39, 95% CI 1.82–3.14) and PFS (HR 2.12, 95% CI 1.56–2.89). In addition, the results showed that the patients receiving long-term administration of temozolomide did not experience additional toxicity over that of the Stupp regimen (6 cycles of temozolomide). It could be concluded that it's efficacious and safe for HGG patients to receive long-term therapy with temozolomide. Nevertheless, more randomized controlled trials (RCTs) should be carried out to verify this conclusion.

## INTRODUCTION

Glioma is the most common primary tumor in the central nervous system (CNS). It accounts for nearly 80% [1, 2]. The HGG patients have a median survival of 15 months [3]. Currently, the first-line therapy for HGG is gross-total resection, concurrent radiation therapy and temozolomide chemotherapy followed by consecutive 6 cycles (patients received a daily dose of 150-200 mg/m2 for 5 days every 28 days) of adjuvant temozolomide therapy [4]. There are no consistent guidelines world-wide on further treatments for patients experiencing stable disease after the first 6 cycles of temozolomide. However, HGG patients who stopped receiving temozolomide at or before 6 cycles suffered underlying risks of tumor recurrence and mortality [5]. Therefore, some medical centers have attempted to prolong temozolomide administration. Their results have demonstrated the efficacy and safety of the long-term therapy with temozolomide for HGG patients [6–8]. This meta-analysis summarizes the data from several comparative studies and comprehensively evaluates the safety, feasibility, and efficacy of long-term therapy with temozolomide (> 6 cycles) for HGG patients.

## RESULTS

### Study screening and its characteristics

**Figure 1:**
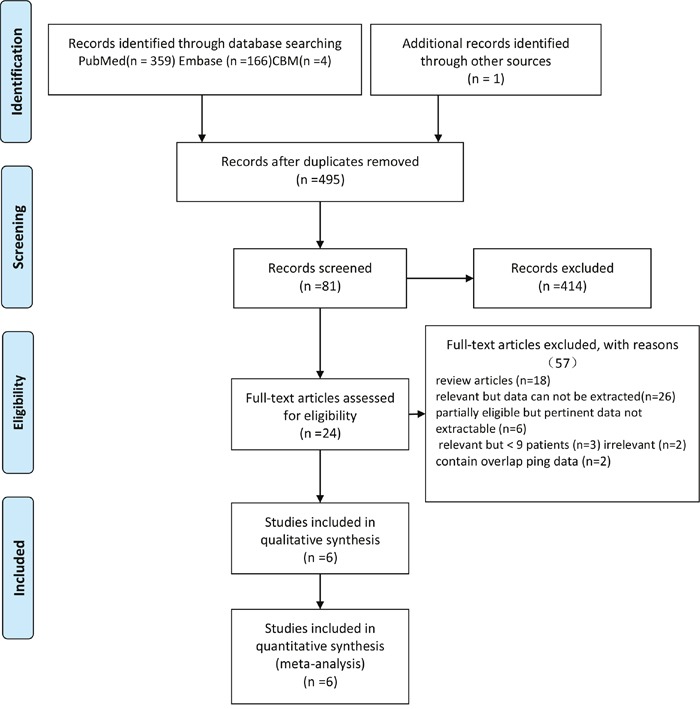
Flow diagram of the study selection process

Searches of pubmed, Embase and Chinese Biomedical databases (CBM) identified 359, 166 and 4 citations, respectively. An additional study was available from the reference lists of eligible studies. After duplication having been removed, 494 records were eligible for further screening by titles and abstracts. Finally, 24 studies were suitable for full-text evaluation. In all, 6 studies [9–14] comprising a total number of 396 cases meeting all inclusion and exclusion criteria were included for the meta-analysis. The sample sizes ranged from 37 to 114. These 396 patients had a mean age of 53.13. The PRISMA flow diagram of the study selection process is displayed in Figure 1. The basic characteristics of all 6 studies are summarized in Table 1.

**Table 1:** Characteristics of studies included in the meta-analysis

Study	Year	Country	Study design	Cases	Cycles of TMZ	Mean age, (years)	KPS at diagnosis	M/F	Histology	RT	Median PFS (months)	Median OS (months)
Seiz	2010	Germany	R	114	6C (55)	62	na	74/40	IV (55)	114	7	15
					Long-term (59)	na			IV (59)			
Freyschlag	2011	Germany	R	42	6C (11)	38.5	na	25/17	III (11)	42	22.2	39
					Long-term (31)				III (31)			
Gloria B.	2012	Canada	R	52	6C (23)	53	90	13/10	IV (23)	23	11.8	16.5
					Long-term (29)	55	80	19/10	IV (29)	29	15.6	24.6
Darlix	2013	France	R	58	6C (38)	56.3	80	28/10	IV (38)	38	18	28.2
					Long-term (20)	52.6	76.7	10/10	IV (20)	20	28.4	30
Barbagallo	2014	Italy	R	37	6C (18)	64.8	62.2	9/9	IV (18)	18	4	8
					Long-term (19)	56.1	71.5	10/9	IV (19)	19	20	28
Weilin	2016	China	R	93	6C (48)	50	86.7	29/19	III (23);IV (25)	45	21	28
					Long-term (45)	43	85.4	34/11	III (16);IV (29)	48	29	39

R: respective; P: prospective; M: male; F: female; TMZ: temozolomide; 6C: 6 cycles of temozolomide. KPS: Karnofsky performance status; RT: radiotherapy; PFS: progress free survival; OS: overall survival.

Of these included studies, 6 were enrolled in the pooled HR analysis of OS, and 4 studies were included in the pooled HR analysis of PFS. In addition, the adverse events from each study were analyzed and displayed in Table 2.

**Table 2:** A toxicity comparison between 6C and long-term groups

Study	Year	6C events	6C totals	Long-term events	Long-term totals
Seiz	2010	33	55	10	59
Freyschlag	2011	7	11	5	31
Gloria B.	2012	na	23	na	29
Darlix	2013	3	38	2	20
Barbagallo	2014	4	18	0	19
Weilin	2016	20	48	0	45

6C: 6 cycles of temozolomide.

**Figure 2:**
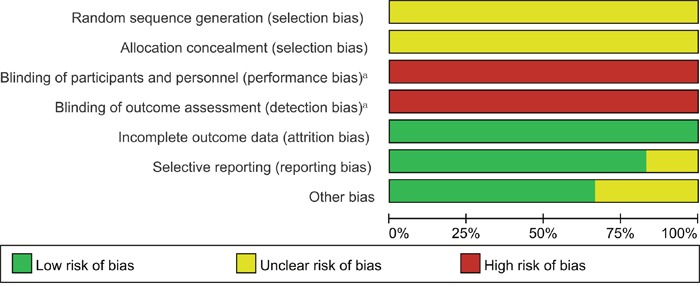
Risk of bias percentile chart

We qualitatively judged the quality test of each study using the standard Cochrane Collaboration's tool, and the summary analysis is shown in Figure 2. All 6 studies included were nonrandomized studies, which were considered to have low risk despite the lack of double blinding. The main patient characteristics (age, gender, Karnofsky performance status, extent of excision) showed no significant differences among these studies.

### Efficacy

**Figure 3:**
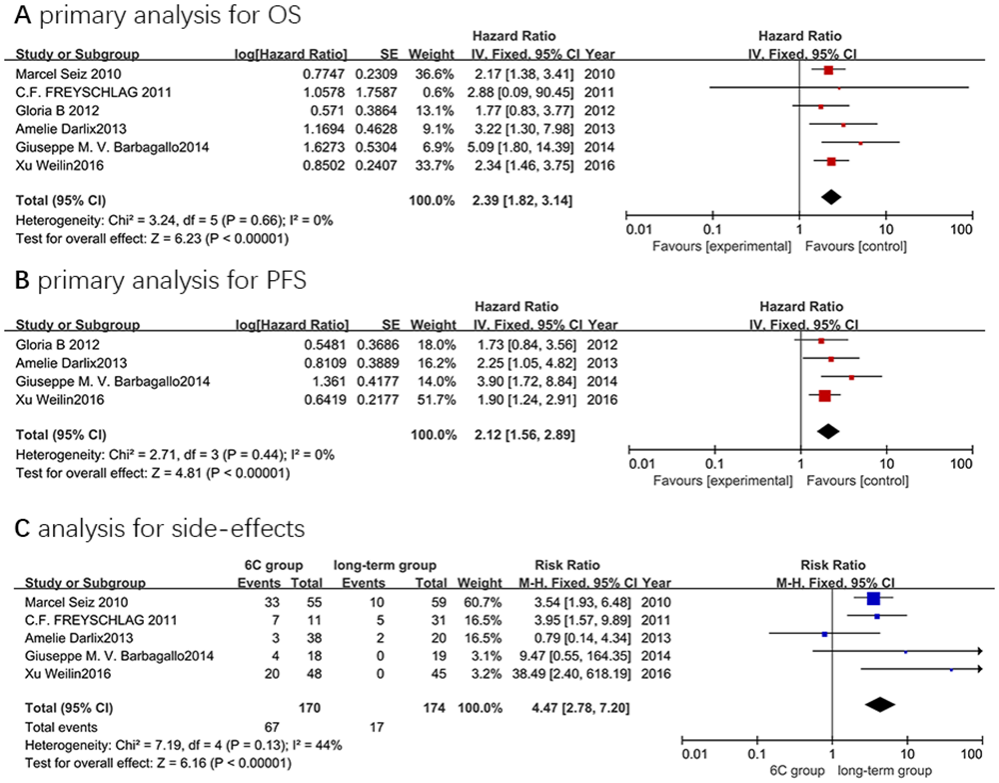
Forest plot of comparison: 6C group versus long-term group **(A)** the primary analysis for OS; **(B)** the primary analysis for PFS; and **(C)** the analysis for side-effects.

Six studies were enrolled in the OS analyses. No heterogeneity (x^2^ = 3.24, P = 0.66, I^2^ = 0 %) was observed among these studies (Figure 3A). Therefore, the HR and 95% CI were calculated by the fixed effects model (396 total cases, HR 2.39, 95% CI 1.82–3.14). The results demonstrated a significant reduction in the risk of death in patients receiving long-term administration of temozolomide. The median OS from the included studies is 22.93 months for the 6C group versus 27.65 months for the long-term group.

Four studies were included in the PFS analyses. There was no heterogeneity (x^2^ = 2.71, P = 0.44, I^2^ = 0 %) among these four studies (Figure 3B). Hence, the fixed effects model (240 total cases, HR 2.12, 95% CI 1.56–2.89) was applied in the HR pooled analyses. This meta-analysis suggested that the long-term regimen was superior to the Stupp regimen (6 cycles of temozolomide) in reducing the risk of tumor recurrence. Besides, the median PFS from the included studies is 15.7 months for the 6C group versus 21.25 months for the long-term group. However, the results should be interpreted with caution due to the limited data.

### Safety

We could not derive adverse effects data from the report of Gloria B et al. So there were five studies included in the safety analyses. We analyzed total toxicity events from each study (displayed in Table 2) without further subgroup analyses due to the limited data. No heterogeneity (x^2^ = 7.19, P = 0.13, I^2^ = 44 %) was observed among these studies (Figure 3C). Thus, the fixed effects model (84 total events, RR 4.47, 95% CI 2.78–7.20) was employed in the RR pooled analyses. The results showed that patients receiving long-term administration of temozolomide did not experience additional toxicity over that of Stupp regimen (6 cycles of temozolomide). Furthermore, most of the adverse effects could be managed by reducing the dose temporally or delaying the next cycle.

### Publication bias

The Deek's funnel plot and Egger's rank correlation test demonstrated that there was no publication bias across the included studies regarding OS and PFS (p = 0.358, p = 0.419, respectively).

## DISCUSSION

For HGG patients experiencing well to the Stupp regimen (6 cycles of temozolomide), further treatments are warranted in preventing recurrence or progression. Although several medical centers had attempted to prolong the administration of temozolomide, there are only several case reports and small series available in the literature. Besides, long-term therapy with temozolomide remains controversial due to a lack of clinical guideline. This meta-analysis includes six studies and comprehensively evaluates the safety, feasibility, and efficacy of long-term therapy with temozolomide (>6 cycles) for HGG patients.

Temozolomide is a second generation of alkylating agent which breaks the DNA double-strand, thus causing cell death [15]. Temozolomide is a better tolerated agent than other chemotherapeutic agents and is clinically widely used.

This meta-analysis has included six studies comprising 396 patients in the pooled analysis. The results of quantitative synthesis demonstrated that long-term therapy with temozolomide is superior to the Stupp regimen. HGG patients achieved longer OS (median:22.93 versus 27.65 months; HR 2.39, 95% CI 1.82–3.14) and PFS (median:15.7 versus 21.25 months; HR 2.12, 95%CI 1.56–2.89) when they received long-term temozolomide. In addition, there was no heterogeneity and publication bias in the pooled analysis of OS and PFS, further bolstering the statistical reliability of the results of this meta-analysis.

In addition to the six studies included, several single-arm studies have also reported an efficacy of long-term regimen for HGG patients [16–18]. Besides, Doo-Sik Kong and his colleagues investigated the prognostic impact of several molecular phenotypes in 58 HGG patients who received extended administration of temozolomide. Their results suggested that the isocitrate dehydrogenase-1 (IDH1) mutation displayed the greatest impact on the therapeutic effects of temozolomide [19]. MGMT is a DNA-repair protein that it can counter the effect of temozolomide by removing alkyl groups from guanine, allowing cancer cells to be resistant to temozolomide chemotherapy [20]. temozolomide could exert its greatest effects in patients with a methylated O6-methylguanine-DNA methyltransferase (MGMT) promoter, by killing sensitive tumor cells [1, 22]. It has also been reported that MGMT methylation improved the efficacy of temozolomide [23]. As a result, patients with methylated MGMT may benefit more from long-term therapy with temozolomide, which has been demonstrated in several studies [13, 23].

The results regarding toxicity showed that patients receiving long-term temozolomide did not experience additional toxicity over the Stupp regimen (6 cycles of temozolomide). The report of Seiz et al. suggested that for patients receiving long-term administration of temozolomide, treatments had to be stopped less frequently due to drug-related toxicity (HR 0,909) compared to the Stupp regimen [9]. The conclusion is also consistent with several other single-arm studies. A. Berrocal. P and his colleagues increased the dose of temozolomide to 1785 mg/m^2^/cycle compared to the commonly used regimen (1000 mg/m^2^/cycle). Their results suggested that the patients could benefit from this regimen with manageable toxicity [24]. The common adverse effects were reported to be hematotoxicity and gastrointestinal toxicity. However, most of these adverse effects could be managed by reducing the dose temporally or delaying the next cycle. From what had been reported, patients undergoing long-term temozolomide chemotherapy exhibited low incidence of severe toxicity (CTC grade 3 or 4 toxicity). And increasing the number of cycles did not lead to additional temozolomide related side effects. Mustafa Khasraw once reported a patient who received 98 cycles of temozolomide chemotherapy [25]. Others reported the number of cycles as many as 101 cycles [13]. All the data above indicate the safety and feasibility of long-term temozolomide treatment in HGG patients.

### Limitations

There some limitations in our meta-analysis.

Firstly, only six non-randomized studies have been included. So the results should be interpreted cautiously due to the limited data although the results of this meta-analysis are robust.

Secondly, the basis for grouping patients in each study is slightly different. That could have an impact on the OS and PFS in each study to some degrees.

Thirdly, although there was no statistical public bias in the overall analysis, only papers published in English and Chinese with full-text were included in this meta-analysis. This may leave out other eligible studies that were unpublished or reported in other languages.

Fourthly, all the studies included were retrospective. So, the patients in long-term group may be more likely to tolerated well of temozolomide, which may introduce bias in patient selection. Therefore, more RCTs should be carried out to verify this conclusion.

## MATERIALS AND METHODS

### Search strategy

We conducted this meta-analysis according to the PRISMA guidelines (Supplementary Table 1). We systematically searched the pubmed, Embase and Chinese Biomedical (CBM) databases for eligible studies. The search time was from database inception to January 1, 2017, with language restricted to English and Chinese. We used the combination of free-text words and MeSH terms as follows: (glioma/glioblastoma/brain neoplasm/brain tumor) AND (temozolomide/temozolamide/temodar) AND (long-term/prolonged/extended). Reference lists from eligible studies were also searched thoroughly for potential relevant studies.

The selection process of eligible studies was performed by two independent authors (W.l. Xu and L.S. Gao)

### Inclusion and exclusion criteria

The inclusion criteria are as follows: (1) Randomized controlled trials (RCTs) or other comparative studies; (2) Temozolomide was used to treat HGG patients; (3) At least one group of patients had received a median period of more than 6 cycles of temozolomide; (4) The data on overall survival, progression free survival and toxicity could be extracted from each included study; (5) At least 10 patients were included in each study; (6) There were no overlapping data.

The exclusion criteria are as follows: (1) The study did not meet the inclusion criteria; (2) Reviews, letters, editorials, abstracts, case reports, congresses and communications; (3) Single-arm studies.

### Data extraction and quality assessment

Data of interest were extracted as follows: (1) identity: authors, years, country; (2) type of design: prospective or retrospective, RCTs or comparative studies; (3) patinets included in each study: age, gender, histology, Karnofsky Performance Status (KPS) at diagnosis; (4) treatments: schedules of radiotherapy and chemotherapy; (5) outcomes: OS, PFS, incidence of toxicity.

The related data from eligible studies were collected and summarized by two of the authors, respectively (W.l. Xu and T. Li). Any discordance was settled by a third author (J.M. Zhang).

The methodological quality of each study was assessed by using the domain-based Cochrane Collaboration's tool [26]. Any dispute was resolved by a third author (A.W. Shao).

### Types of outcome measurements

#### Efficacy indicators

Overall survival (OS) was measured from the time of resection to patient death or the last date when the patient was known to be alive. Progress free survival (PFS) was defined as the time from resection to the time of demonstrated tumor growth on follow-up imaging according to the MacDonald criteria, or evidence of neurological decline.

#### Safety indicators (adverse events)

toxicity was based on National Cancer Institute Common Terminology Criteria (NCI-CTC).

### Statistical methods

This meta-analysis was completed based on the Review Manager Version 5.0 software (The Cochrane Collaboration, Software Update, Oxford, UK), which was provided by the Cochrane Collaboration, and Stata 14. The efficacy was assessed by using pooled HRs, along with its 95% confidence interval (CI) for dichotomous variables, for OS and PFS, with a value >1 indicating the advantage of long-term therapy with temozolomide. The safety was evaluated by using pooled RR, along with its 95% confidence interval (CI) for dichotomous variables, with a value >1 indicating that the patients receiving more than 6 cycles of temozolomide did not increase the risks of drug-related toxicity.

The heterogeneity from each study was calculated by chi-squared value test and inconsistency index (I^2^). Significant heterogeneity was identified with a value of P < 0.1 or I^2^> 50%, then random effect model was adopted. Otherwise, the fixed effect models were used. We performed meta-regression or subgroup analyses to find the source of heterogeneity [27, 28]. If it is necessary, sensitivity analyses were also performed.

Publication bias was evaluated by Deek's funnel plot visually and analytic methods (Egger's test) using Stata14.0 (StataCorp LP, College Station, TX) [29, 30]. The value of P < 0.05 indicates significant asymmetry [31].

## CONCLUSION

This meta-analysis has demonstrated the superiority of long-term therapy with temozolomide in selected patients. Furthermore, long-term administration of temozolomide did not add additional toxicity to the HGG patients over the Stupp regimen (6 cycles of temozolomide). So it's efficacious and safe for HGG patients to receive long-term therapy with temozolomide. Nevertheless, more RCTs should be carried out to verify this conclusion.

## SUPPLEMENTARY MATERIALS FIGURES AND TABLES





## Abbreviations

HGG: high-grade glioma
CBM: Chinese Biomedical
OS: overall survival
PFS: progression free survival
HR: hazard ratio
RR: risk ratio
RCTs: randomized controlled trials
CNS: central nervous system
KPS: Karnofsky performance status
IDH1: isocitrate dehydrogenase-1
MGMT: methylated O6- methylguanine-DNA methyltransferase
NCI-CTC: National Cancer Institute Common Terminology Criteria
CI: confidence interval
