# Supplementary material for: Efficacy and safety of long-term therapy for high-grade glioma with temozolomide: A meta-analysis
Source: Oncotarget. 2017 Apr 24;8(31):51758–65. doi: 10.18632/oncotarget.17401 (PMC5584285; doi:10.18632/oncotarget.17401)
Supplement: Supplementary file 1 [file oncotarget-08-51758-s001.pdf]

Efficacy and safety of long-term therapy for high-grade glioma with temozolomide: A meta-analysis

SUPPLEMENTARY MATERIALS

Supplementary Table 1: PRISMA checklist

See Supplementary File 1
